# Glutamate regulates gliosis of BMSCs to promote ENS regeneration through α-KG and H3K9/H3K27 demethylation

**DOI:** 10.1186/s13287-022-02936-7

**Published:** 2022-06-17

**Authors:** Mengke Fan, Huiying Shi, Hailing Yao, Weijun Wang, Yurui Zhang, Chen Jiang, Rong Lin

**Affiliations:** Department of Gastroenterology, Union Hospital, Tongji Medical College, Huazhong University of Science and Technology, Wuhan, 430022 China

**Keywords:** Bone marrow-derived mesenchymal stem cells (BMSCs), α-ketoglutarate (α-KG), Glud1 (glutamate dehydrogenase 1), Histone methylation

## Abstract

**Background:**

There is a lack of effective therapies for enteric nervous system (ENS) injury. Our previous study showed that transplanted bone marrow-derived mesenchymal stem cells (BMSCs) play a “glia-like cells” role in initiating ENS regeneration in denervated mice. Cellular energy metabolism is an important factor in maintaining the biological characteristics of stem cells. However, how cellular energy metabolism regulates the fate of BMSCs in the ENS-injured microenvironment is unclear.

**Methods:**

The biological characteristics, energy metabolism, and histone methylation levels of BMSCs following ENS injury were determined. Then, glutamate dehydrogenase 1 (Glud1) which catalyzes the oxidative deamination of glutamate to α-KG was overexpressed (OE) in BMSCs. Further, OE-Glud1 BMSCs were targeted–transplanted into the ENS injury site of denervated mice to determine their effects on ENS regeneration.

**Results:**

In vitro, in the ENS-injured high-glutamate microenvironment, the ratio of α-ketoglutarate (α-KG) to succinate (*P* < 0.05), the histone demethylation level (*P* < 0.05), the protein expression of glial cell markers (*P* < 0.05), and the gene expression of Glud1 (*P* < 0.05) were significantly increased. And the binding of H3K9me3 to the GFAP, S100B, and GDNF promoter was enhanced (*P* < 0.05). Moreover, α-KG treatment increased the monomethylation and decreased the trimethylation on H3K9 (*P* < 0.01) and H3K27 (*P* < 0.05) in BMSCs and significantly upregulated the protein expression of glial cell markers (*P* < 0.01), which was reversed by the α-KG competitive inhibitor D-2-hydroxyglutarate (*P* < 0.05). Besides, overexpression of Glud1 in BMSCs exhibited increases in monomethylation and decreases in trimethylation on H3K9 (*P* < 0.05) and H3K27 (*P* < 0.05), and upregulated protein expression of glial cell markers (*P* < 0.01). In vivo, BMSCs overexpressing Glud1 had a strong promotion effect on ENS regeneration in denervated mice through H3K9/H3K27 demethylation (*P* < 0.05), and upregulating the expression of glial cell protein (*P* < 0.05).

**Conclusions:**

BMSCs overexpressing Glud1 promote the expression of glial cell markers and ENS remodeling in denervated mice through regulating intracellular α-KG and H3K9/H3K27 demethylation.

**Supplementary Information:**

The online version contains supplementary material available at 10.1186/s13287-022-02936-7.

## Background

Gastrointestinal motility disorders are important digestive system diseases, and its incidence has increased in recent years [1]. Enteric nervous system (ENS) injury is the important pathogenic factor [2]. According to previous studies, nerve regeneration after ENS injury in adults is difficult [3]. There is a lack of effective treatment for ENS regeneration. However, bone marrow-derived mesenchymal stem cells (BMSCs) show promising potential to repair damaged nerves in multiple nerve injury models [4, 5]. Our previous studies showed that BMSCs could survive in the gastrointestinal microenvironment and promote ENS regeneration and functional repair following ENS injury [6, 7].

Cellular energy metabolism is an important factor in maintaining the biological characteristics of stem cells. The tricarboxylic acid (TCA) cycle plays a key role in cell metabolism and is closely associated with various diseases [8, 9]. Glutamate is an essential substance in TCA and provides energy for cell growth. Glutamate can regulate neurogenesis, neurite outgrowth, and neuron survival in the nervous system [10, 11]. In addition, glutamate can be dehydrogenated to α-ketoglutarate (α-KG) in glial cells during TCA energy metabolism [12]. Carey et al. reported that intracellular α-KG maintains the pluripotency of embryonic stem cells (ESCs) [13]. In the absence of exogenous glutamine, naive ESCs cells exhibited an elevated α-KG-to-succinate ratio promoting histone demethylation.

Histone demethylase uses α-KG as a co-substrate to remove methyl groups on histones. Histone modification has been reported to affect stem cell biological processes such as differentiation and aging [14]. Transcriptomics studies showed that histone demethylases were closely related to the self-renewal of ESCs [15]. In addition, other studies showed that histone methylation could affect neurogenesis and differentiation [16, 17]. During neural development, the H3K27 demethylase can activate specific components affecting normal brain development in zebrafish [18]. Fiszbein et al*.* reported that regulating the efficiency of H3K9 histone methylation can affect neuronal differentiation [19]. However, how histone methylation regulates the fate of BMSCs in the ENS-injured microenvironment is unclear.

Our previous study showed that the transplanted BMSCs play a “glia-like cells” role to initiate nerve regeneration in ENS injury. Studies have shown that histone methylation plays an important role in the maintenance of glial cell phenotype. Glial fibrillary acidic protein (GFAP) is expressed by glial cells, which plays a significant role in maintaining the structure and function of glial cells and repairing nervous system damage [20, 21]. The level of histones methylation can affect the binding of GFAP regulator STAT/CBP to the promoter region, thereby affecting the expression of GFAP [22, 23]. In addition, a change in the methylation level of the GFAP promoter could also affect GFAP expression [24]. However, further research is needed to determine how cellular energy metabolism regulates the glial cell characteristics of BMSCs.

This study aimed to explore how cellular energy metabolism regulates the fate of BMSCs to promote ENS regeneration in the ENS injury microenvironment and thus further promote ENS remodeling.

## Methods

### Animals

Eight-week-old wild-type C57BL/6 male mice (22 ± 2 g) were purchased from Beijing Huafukang Biosciences Co., Ltd. They were housed under a specific pathogen-free (SPF) laboratory with free access to food and water. All experimental procedures were performed in accordance with the Animal Ethics Committee of Tongji Medical College of Huazhong University of Science and Technology.

### BMSCs  culture and treatment

Bone marrow-derived mesenchymal stem cells (BMSCs) were isolated as previously described [6]. They were cultured in low-glucose Dulbecco’s modified eagle medium (DMEM) supplemented with 10% fetal bovine serum (FBS) at 37˚C in 5% CO_2_. When the cell confluence reached 80%, glutamate (Glu, Sigma, 2 mM), dimethyl-alpha-KG (DM-α-KG, Sigma, 4 mM), dimethyl succinate (DM-Suc, Topscience, 4 mM), or D-2-hydroxyglutarate (D-2HG, Cayman, 2 mM) was added to the medium [25]. The cells were harvested for various assays after incubation.

### Lentivirus transfection

Lentiviral vectors (Genomeditech, Shanghai, China) expressing glutamate dehydrogenase 1 (Glud1)-specific RNA, including Glud1-knockdown (KD), Glud1-overexpression (OE), and Glud1-negative control (NC), expressing green fluorescent protein (GFP) were constructed. Recombinant lentiviruses were added at a cell density of 60%. The supernatant was discarded 24 h after infection and replaced with a complete fresh medium. A microscope was used to observe the cells and predict transfection efficiency.

### Grouping and BMSCs transplantation

The mice were randomly divided into four groups (*n* = 6 per group): (1) control group, (2) benzalkonium chloride (BAC) group, (3) BAC mice injected with BMSCs-NC group (BAC + BMSCs-NC); (4). BAC mice injected with BMSCs-OE group (BAC + BMSCs-OE). The gastric denervation model was constructed using 0.05% BAC (Merck, CAS: 63449-41-2). A midline incision was made on the mice. Thereafter, a 1-cm segment of the gastric tissue was wrapped with gauze and soaked in BAC for 15 min. However, saline was used for the control groups. Transplantation of the BMSCs was done 3 days after the denervation model was successfully established. The BMSCs were transplanted into denervated gastric serosal surfaces using a 22-gauge needle. The BMSCs were preconditioned using neurotrophic factors: glial cell line-derived neurotrophic factor (GDNF), basic fibroblast growth factor (b-FGF), and epidermal growth factor (EGF) (10 ng/ml) for 10 days before the transplantation. All mice were killed 4 weeks after injection with the BMSCs. The tissues were then collected and analyzed.

### Cell proliferation and migration

Cells (1 × 10^6^/ml) were incubated in PBS containing CFSE (10 μM) at 37 °C for 10 min. The reaction was then quenched with DMEM. After washing with PBS three times, the fluorescence of the cells in each group was determined using flow cytometry. Transwell assay (pore size, 8 μm; Corning Inc., Corning, NY, USA) was used for cell migration assay. A total of 1 × 10^5^ cells were seeded on the upper chamber. Following incubation for 24 h, the Transwell migration system was stained with crystal violet and observed under a microscope (Olympus Corporation).

### Detection of the metabolites

Glutamate (Jiancheng, Nanjing, China), α-KG (BioVision, USA), and succinate (BioVision, USA) were quantified using commercial kits. Targeted metabolic assays were performed according to the protocols.

### RNA extraction and real-time quantitative PCR (qPCR)

Total RNA was extracted using TRIzol reagent (Vazyme) in accordance with the protocol and transcribed into cDNA with a cDNA synthesis kit (Takara Bio). Real-time RT-PCR was performed with StepOne Real-Time PCR system (Applied Biosystems) and normalized to GAPDH using the △△Ct method. The primers sequences are listed in Additional file 1: Table S1.

### Chromatin immunoprecipitation assays

Chromatin immunoprecipitation (ChIP) assays (LOT:26156, Thermo) were conducted according to the manufacturer’s instructions. Antibody against H3K9me3 was obtained from CST. ChIP-DNA was amplified by qPCR using SYBR Green PCR Master Mix (Takara Bio). Results were normalized to input DNA, and the primers are listed in Additional file 1: Table S1.

### Enzyme-linked immunosorbent assay (ELISA)

The supernatant from BMSCs in different groups was collected and centrifuged at 2000 × *g* for 20 min. The secretion level of GDNF and S100B was measured using ELISA kits (Cloud-Clone Corp., USA) according to the manufacturer’s instructions.

### Protein preparation and western blot analysis

The total proteins and nuclear proteins were extracted from the cells or tissue samples using a radioimmunoprecipitation assay (RIPA) buffer supplemented with phenylmethylsulfonyl fluoride (PMSF). Protein concentration was determined using bicinchoninic acid (BCA) protein assay kit (Vazyme, China). The proteins were then separated using SDS-PAGE and then electro-transferred to polyvinylidene difluoride (PVDF) membranes. After soaking in 10% skimmed milk powder for 1 h, the membranes were incubated overnight at 4 °C with specific antibodies: GFAP (ABclonal, Wuhan, China), GDNF (Abcam, Cambridge, UK), S100B (Abcam, Cambridge, UK), GAPDH (Antgene, Wuhan, China), H3K9me1 (A2358, ABclonal), H3K9me3 (A2360, ABclonal), H3K27me1 (A2361, ABclonal), H3K27me3 (A2363, ABclonal), and H3 (A2348, ABclonal). Thereafter, the membranes were incubated at room temperature with HRP-labeled secondary antibodies for 1 h. The intensities of the protein bands were determined using a chemiluminescence (ECL) kit (Vazyme).

### Immunofluorescence

For the myenteric plexus, fresh gastric tissues were placed in pre-cooled phosphate-buffered saline (PBS) solution. Thereafter, the mucosa and muscularis tissues were separated and fixed in 4% paraformaldehyde for 10 min. The paraffin-embedded gastric tissue sections were dewaxed and hydrated and subjected to antigen retrieval. The tissues were incubated with donkey serum containing 0.3% Triton X‐100 at 4 °C overnight for blocking of nonspecific binding. Subsequently, the tissue sections were incubated overnight at 4 °C with the specific primary antibodies: GFAP (ABclonal, Wuhan, China), HuC/D (Abcam, Cambridge, UK), S100B (Abcam, Cambridge, UK), β-Tubulin (ABclonal, Wuhan, China) H3K9me1 (A2358, ABclonal), H3K9me3 (A2360, ABclonal), H3K27me1 (A2361, ABclonal), and H3K27me3 (A2363, ABclonal). After washing three times with PBS, the preparations were then stained with the secondary antibody and incubated for 2 h at room temperature. The cell nuclei were stained with 4′,6-diamidine-2′-phenylindole dihydrochloride (DAPI) for 20 min. The specimens were observed using a confocal laser scanning microscope (Olympus, Tokyo, Japan).

### Statistical analysis

Statistical analysis was conducted using SPSS version 20.0 (IBM Corp.). Further, GraphPad prism software 7.0 (GraphPad Software, Inc.) and Image J software were used to plot the graphs. All experimental data were presented as the mean ± SD. Unpaired Student’s t test was used for comparison between two groups. One-way analysis of variance (ANOVA) was used for comparisons between multiple groups. A *P* value < 0.05 was considered statistically significant.

## Results

### The expression of characteristic glial cell proteins, cell migration, intracellular α-KG and histone demethylation level of BMSCs were increased in the ENS-injured high-glutamate microenvironment

**Fig. 1:**
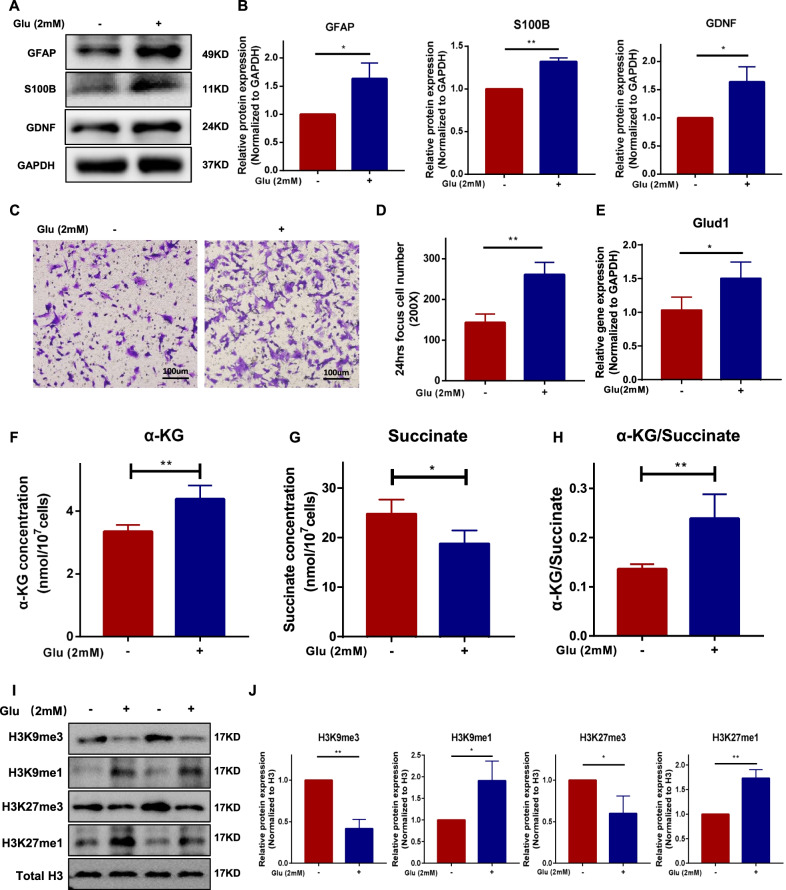
The expression of characteristic glial cell proteins, cell migration, intracellular α-KG, and histone demethylation of BMSCs were increased in homoglutamate microenvironment. Glutamate (2 mM) was incubated with BMSCs for 24 h. **A** and **B** Representative immunoblot bands and histogram of relative expression for the GFAP (Control vs. Glu: 1 vs. 1.63 ± 0.23, *P* < 0.05), S100B (Control vs. Glu: 1 vs. 1.32 ± 0.03, *P* < 0.01), and GDNF (Control vs. Glu:1 vs. 1.64 ± 0.22, *P* < 0.05) proteins. GAPDH was used as a loading control. **C** and **D** Transwell migration experiment of BMSCs and statistical analysis (Control vs. Glu: 140 ± 20.3 vs. 256 ± 27.4, *P* < 0.01). **E** The transcripts of Glud1 were determined by a reverse transcription-polymerase chain reaction (RT-PCR) assay (Control vs. Glu: 1.0 ± 0.18 vs. 1.5 ± 0.22, *P* < 0.05). **F**–**H** The intracellular α-KG (Control vs. Glu: 3.34 ± 0.18 nmol vs. 4.38 ± 0.37 nmol, *P* < 0.01), succinate (Control vs. Glu: 24.7 ± 2.6 nmol vs. 18.7 ± 23 nmol, *P* < 0.05), concentration and α-kg/succinate ratio (Control vs. Glu: 0.13 ± 0.01 vs. 0.24 ± 0.05, *P* < 0.01) of BMSCs. **I** and **J** Representative immunoblot bands and histogram of relative expression for the H3K9me3 (Control vs. Glu: 1 vs. 0.42 ± 0.09, *P* < 0.01), H3K9me1 (Control vs. Glu: 1 vs. 1.90 ± 0.37, *P* < 0.05), H3K27me3 (Control vs. Glu: 1 vs. 0.60 ± 0.17, *P* < 0.05), and H3K27me1 (Control vs. Glu: 1 vs. 1.7 ± 0.14, *P* < 0.01) proteins. H3 was used as a loading control. Glu: glutamate; α-KG: alpha-ketoglutarate; Glud1: glutamate dehydrogenase 1. These results are representative of at least three times independent experiments. *P < 0.05, **P < 0.01

In vivo, the glutamate levels of gastric tissue in ENS injury groups (BAC model) showed an approximately 1.8-fold increase compared with the control groups (*P* < 0.01, Additional file 2: Fig. S1). Different concentrations of glutamate were incubated with BMSCs for 24 or 48 h to explore the effect of glutamate on the biological characteristics of BMSCs (Additional file 2: Fig. S2A–D). The results showed that the protein expression of glial cell characteristic markers (GFAP/GDNF/S100B) was significantly upregulated in the glutamate-exposed group (2 mM, 24 h) compared with controls (Fig. 1A, B, *P* < 0.05). In addition, the migration ability of BMSCs was significantly increased after glutamate intervention (Fig. 1C, D, *P* < 0.01). And the mesenchymal genes (Snail and Twist) and cell migration-related genes (CXCR4) of BMSCs were activated exposed to glutamate (*P* < 0.05, Additional file 2: Fig. S4). Besides, the γ-aminobutyric acid receptors (GABARA) of BMSCs were activated exposed to glutamate (*P* < 0.05, Additional file 2: Fig. S3). However, there were no significant differences in cell proliferation ability between the control and glutamate-exposed groups (Additional file 2: Fig. S2E, F, *P* > 0.05). These results showed that the expression of characteristic glial cell proteins for BMSCs was significantly upregulated in the ENS-injured high-glutamate microenvironment.

Glutamate is a key substrate of the TCA cycle. Therefore, the TCA energy metabolism of BMSCs in the homoglutamate microenvironment was analyzed. The results showed that intracellular α-KG content and the ratio of α-KG/succinate of BMSCs were significantly increased (*P* < 0.01, Fig. 1F, H), and intracellular succinate was decreased in glutamate-exposed groups (*P* < 0.05, Fig. 1G). Glutamate dehydrogenase 1 (Glud1) is key in glutamate metabolism and catalyzes the oxidative deamination of glutamate to α-KG. Besides, the mRNA expression of Glud1 for BMSCs was significantly upregulated in glutamate-exposed groups (*P* < 0.05, Fig. 1E). Histone demethylase relies on α-KG as a co-substrate of demethylation. Thus, we further analyzed the effect of the high-glutamate microenvironment on the histone methylation level of the BMSCs. The result indicated that there was an increased expression in monomethylation and decreased expression in trimethylation on H3K9 and H3K27 (*P* < 0.05, Fig. 1I, J) in glutamate-exposed group.

### The metabolism changes of BMSCs affect the expression of characteristic glial cell proteins and histone demethylation level

**Fig. 2:**
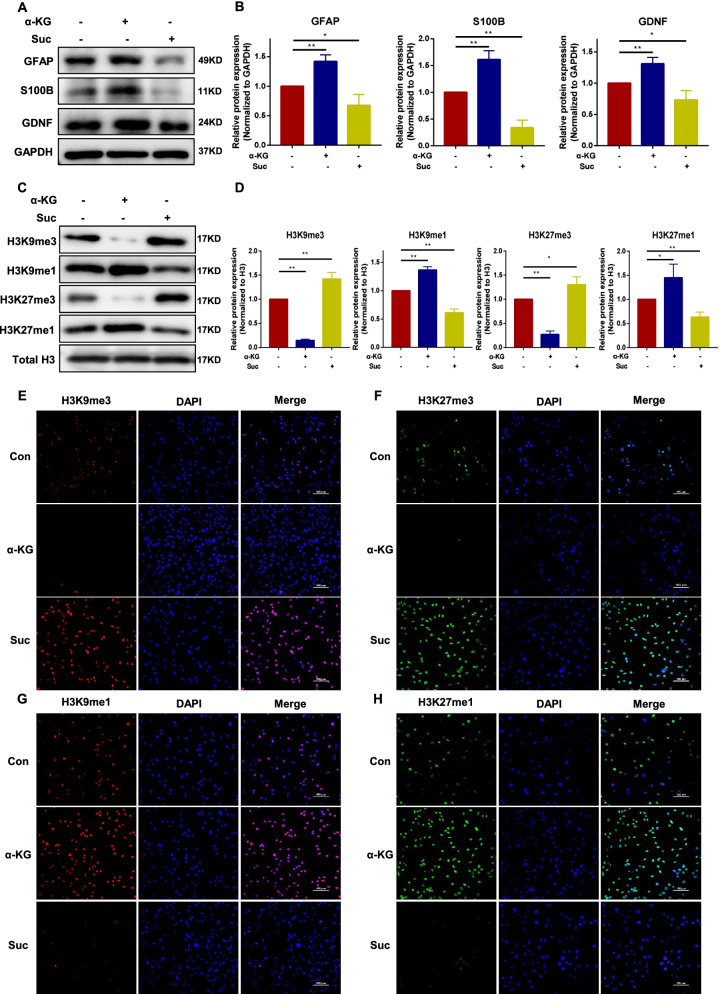
α-KG increases the expression of characteristic glial cell proteins and demethylation of BMSCs; succinate inhibits the expression of characteristic glial cell proteins and demethylation of BMSCs. DM-α-KG (4 mM) or DM-succinate (4 mM) were incubated with BMSCs for 24 h. **A** and **B** Representative immunoblot bands and histogram of relative expression for the GFAP (Control:1, α-KG: 1.42 ± 0.09, Suc:0.68 ± 0.15), S100B (Control:1, α-KG: 1.61 ± 0.13, Suc:0.34 ± 0.11), and GDNF (Control:1, α-KG: 1.31 ± 0.08, Suc:0.73 ± 0.12) proteins. GAPDH was used as a loading control. **C** and **D** Representative immunoblot bands and histogram of relative expression for the H3K9me3(Control:1, α-KG: 0.15 ± 0.02, Suc:1.42 ± 0.11), H3K9me1(Control:1, α-KG: 1.36 ± 0.05, Suc:0.61 ± 0.05), H3K27me3 (Control:1, α-KG: 0.27 ± 0.06, Suc:1.3 ± 0.13), and H3K27me1 (Control:1, α-KG: 1.45 ± 0.23, Suc:0.63 ± 0.09) proteins. H3 was used as a loading control. **E**–**H** Representative immunofluorescence images for H3K9me3 (red), H3K9me1 (red), H3K27me3 (green), and H3K27me1 (green) of BMSCs in each group, the nuclei were labeled with DAPI (blue). Con: control; α-KG: alpha-ketoglutarate; Suc: succinate. These results are representative of at least three times independent experiments. *P < 0.05, **P < 0.01

The BMSCs were incubated with DM-α-KG or DM-succinate to further evaluate the role of the TCA circulating energy metabolism in BMSCs during ENS regeneration. The western blotting results showed that the protein expression level of glial cells characteristic markers (GFAP/S100B/GDNF) was significantly unregulated in the α-KG-exposed group (*P* < 0.01) and downregulated in the succinate-exposed group (*P* < 0.05) when compared with the control group (Fig. 2A, B). Moreover, α-KG intervention increased the monomethylation and decreased the trimethylation on H3K9 (*P* < 0.01) and H3K27 (*P* < 0.05) in BMSCs, which was reversed by the succinate (H3K9: *P* < 0.01; H3K27: *P* < 0.05) (Fig. 2C, D). The immunofluorescence assay showed similar results to the western blot analysis (Fig. 2E–H). All these results indicated that the change of TCA cycle energy metabolism (α-KG/succinate) can affect the expression of characteristic glial cell protein and histone demethylation level of BMSCs.

### H3K9me3 associates with the gene promoter regions of GFAP, S100B, and GDNF. Histone demethylase inhibitor D-2HG alters the protein expression of glial cell marker for BMSCs

**Fig. 3:**
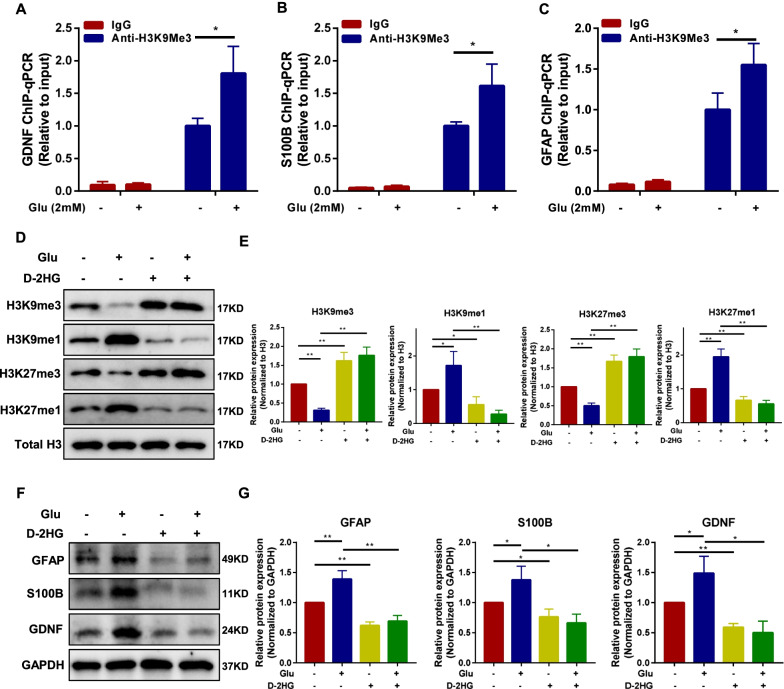
Increased binding of H3K9me3 to the GFAP, S100B, and GDNF promoter in the hyperglutamate environment. Histone demethylase inhibitor D-2HG downregulates the expression of characteristic glial cell proteins for BMSCs. **A–C** Association of H3K9me3 with GFAP, S100B, and GDNF was assessed by chromatin immunoprecipitation (ChIP) assays. DNA was immunoprecipitated with the specific antibody H3K9me3. Bars represent the relative levels of the PCR product of the GDNF (Control vs. Glu: 1 ± 0.10 vs. 1.81 ± 0.34, *P* < 0.05), S100B (Control vs. Glu: 1 ± 0.05 vs. 1.62 ± 0.27, *P* < 0.05) and GFAP (Control vs. Glu: 1 ± 0.17 vs. 1.54 ± 0.21, *P* < 0.05) promoter region’s association with H3K9me3. Non-immunized serum (IgG) was used as a control. Each qPCR assay was repeated two times. Values are the mean ± SD from three technical replicates. BMSCs were incubated with D-2HG (2 mM) or Glu (2 mM) for 24 h. **D** and **E** Representative immunoblot bands and histogram of relative expression for the H3K9me3 (Control:1, Glu: 0.31 ± 0.04, D-2HG: 1.62 ± 0.18, Glu + D-2HG: 1.76 ± 0.18), H3K9me1 (Control:1, Glu: 1.71 ± 0.34, D-2HG: 0.56 ± 0.19, Glu + D-2HG: 0.28 ± 0.10), H3K27me3 (Control:1, Glu: 0.50 ± 0.06, D-2HG: 1.67 ± 0.13, Glu + D-2HG: 1.79 ± 0.17), and H3K27me1 (Control:1, Glu: 1.95 ± 0.19, D-2HG: 0.66 ± 0.09, Glu + D-2HG: 0.56 ± 0.08) proteins. H3 was used as a loading control. **F** and **G** Representative immunoblot bands and histogram of relative expression for the GFAP (Control:1, Glu: 1.39 ± 0.11, D-2HG: 0.62 ± 0.05, Glu + D-2HG: 0.69 ± 0.08), S100B (Control:1, Glu:1.38 ± 0.19, D-2HG: 0.76 ± 0.11, Glu + D-2HG: 0.66 ± 0.12), and GDNF (Control:1, Glu: 1.49 ± 0.23, D-2HG: 0.59 ± 0.05, Glu + D-2HG: 0.50 ± 0.16) proteins. GAPDH was used as a loading control. Glu: glutamate; D-2HG: D-2-hydroxyglutaric acid; ChIP: chromatin immunoprecipitation. These results are representative of at least three times independent experiments. *P < 0.05, **P < 0.01

Association of H3K9me3 with GFAP, S100B, and GDNF was assessed by chromatin immunoprecipitation (ChIP) assays. ChIP assays showed that the association of these genes with H3K9me3 was significantly enhanced upon addition of glutamate (Fig. 3A–C). These data suggested that the glutamate metabolism can regulate the binding of H3K9me3 with GFAP, S100B, and GDNF. D-2-hydroxyglutarate (D-2HG), a competitive inhibitor of α-KG, can be used as an inhibitor of histone demethylation [26]. In this study, BMSCs were treated with D-2HG to determine the role of histone methylation in the protein expression of glial cell markers. The result showed that the treatment of D-2HG significantly upregulated the protein expressions in trimethylation and downregulate the protein expressions in monomethylation on H3K9 and H3K27 (*P* < 0.05, Fig. 3D, E). Moreover, the protein expressions of GFAP, S100B, and GDNF were downregulated in D-2HG-exposed group (*P* < 0.05, Fig. 3F, G). Besides, the intracellular α-KG/succinate of BMSCs following D-2HG treatment was measured. The results showed that the intracellular α-KG content and the ratio of α-KG/succinate of BMSCs were significantly increased upon addition of glutamate (*P* < 0.05). However, the addition of D-2HG reversed this change (*P* < 0.05, Additional file 2: Fig. S5). These results suggest that the binding of H3K9me3 to the GFAP, S100B, and GDNF promoter was significantly enhanced upon addition of glutamate, and inhibition of histone demethylation in BMSCs can alter the expression of characteristic glial cell protein.

### Overexpression of glutamate dehydrogenase 1 (glud1) in BMSCs promotes histone demethylation and upregulates the protein expression of the glial cell markers

**Fig. 4:**
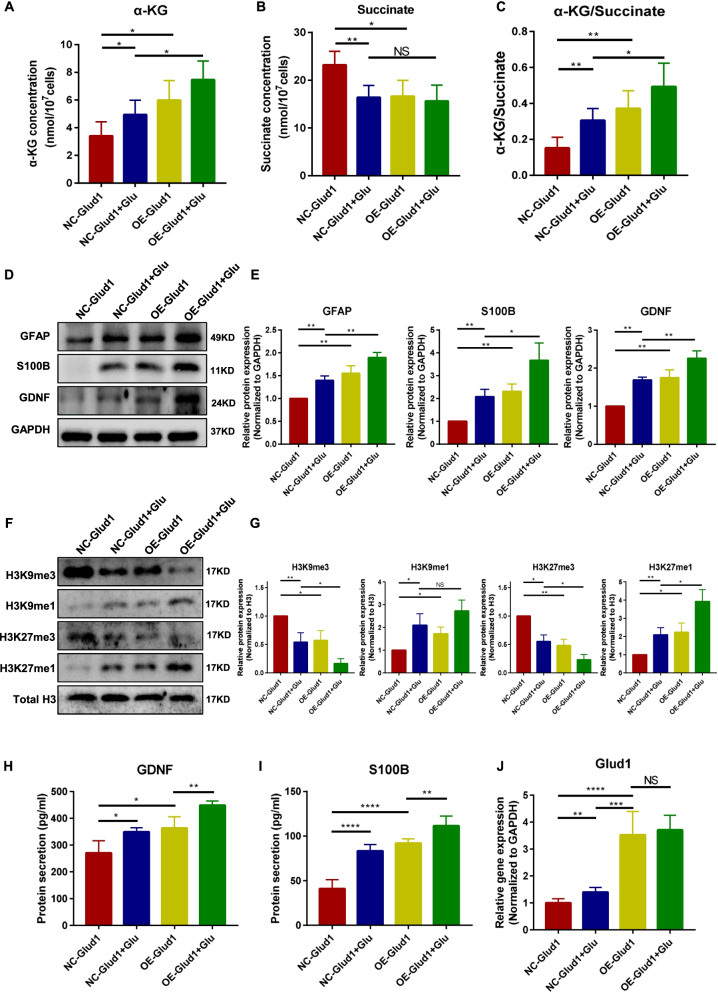
The expression of the characteristic glial cell proteins and histone demethylation was significantly increased in the BMSCs (OE-Glud1) than the BMSCs (NC-Glud1). **A**–**C** The intracellular α-KG (NC: 3.4 ± 0.9 nmol, NC + Glu: 4.9 ± 0.9 nmol, OE: 6.0 ± 1.3 nmol, OE + Glu: 7.4 ± 1.2 nmol), succinate (NC: 23.2 ± 2.6 nmol, NC + Glu: 16.4 ± 2.2 nmol, OE: 16.6 ± 3.0 nmol, OE + Glu: 15.6 ± 3.0 nmol) concentration and α-kg/succinate ratio (NC: 0.15 ± 0.05, NC + Glu: 0.30 ± 0.06, OE: 0.37 ± 0.09, OE + Glu: 0.49 ± 0.12) of BMSCs in different groups. **D** and **E** Representative immunoblot bands and histogram of relative expression of for the GFAP (NC: 1, NC + Glu: 1.40 ± 0.08, OE: 1.55 ± 0.14, OE + Glu: 1.89 ± 0.09), S100B (NC: 1, NC + Glu: 2.08 ± 0.27, OE: 2.31 ± 0.27, OE + Glu: 3.67 ± 0.62), and GDNF (NC: 1, NC + Glu: 1.69 ± 0.06, OE: 1.75 ± 0.17, OE + Glu: 2.26 ± 0.16) in each group. GAPDH was used as a loading control. **F–G** Representative immunoblot bands and histogram of relative expression of for the H3K9me3 (NC: 1, NC + Glu: 0.54 ± 0.13, OE: 0.57 ± 0.14, OE + Glu: 0.17 ± 0.07), H3K9me1 (NC: 1, NC + Glu: 2.10 ± 0.14, OE: 1.73 ± 0.24, OE + Glu: 2.73 ± 0.38), H3K27me3 (NC: 1, NC + Glu: 0.55 ± 0.09, OE: 0.48 ± 0.09, OE + Glu: 0.23 ± 0.07), and H3K27me1(NC: 1, NC + Glu: 2.09 ± 0.33, OE: 2.24 ± 0.41, OE + Glu: 3.93 ± 0.54) in each group. H3 was used as a loading control. **H**–**I** The ELISA level of GDNF (NC: 270 ± 38.98 pg/ml, NC + Glu: 349 ± 13.33 pg/ml, OE: 363.68 ± 36.4 pg/ml, OE + Glu:448.91 ± 13.54 pg/ml) and S100B (NC: 41.05 ± 9.05 pg/ml, NC + Glu: 83.34 ± 6.29 pg/ml, OE: 91.87 ± 4.47 pg/ml, OE + Glu: 111.56 ± 9.77 pg/ml) in the culture medium samples of BMSCs in different groups. **J** The transcripts of Glud1 (NC: 1 ± 0.13, NC + Glu: 1.39 ± 0.15, OE: 3.52 ± 0.77, OE + Glu: 3.71 ± 0.48) were determined by a RT-PCR assay. Glu: glutamate; Glud1: glutamate dehydrogenase 1; OE: overexpression; NC: negative control. These results are representative of at least three times independent experiments. *P < 0.05, **P < 0.01, NS: no significance

Glutamate dehydrogenase 1 (Glud1) is key in glutamate metabolism and catalyzes the oxidative deamination of glutamate to α-KG [27]. Therefore, BMSCs (Glud1-KD) and BMSCs (Glud1-OE) were constructed to determine the role of energy metabolism in BMSCs during ENS regeneration (Additional file 2: Fig. S6A). The protein expression of Glud1 was downregulated in BMSCs (Glud1-KD) (*P* < 0.01, Additional file 2: Fig. S6B) and upregulated (*P* < 0.01, Additional file 2: Figure S6C) in BMSCs (Glud1-OE) when compared with BMSCs (Glud1-NC). The intracellular α-KG, succinate concentration, and α-kg/succinate ratio of BMSCs in different groups were measured. The result indicated that the intracellular α-KG content and the ratio of α-KG/succinate were significantly increased for OE-Glud1 BMSCs (*P* < 0.01, Fig. 4A–C) and decreased for KD-Glud1 BMSCs (*P* < 0.01, Additional file 2: Fig. S6D–F) compared with NC-Glud1 BMSCs. Moreover, the expression of glial cell proteins and the histone methylation level of BMSCs were evaluated. The result showed that knockdown of Glud1 downregulated the expression of glial cell proteins (GFAP, *P* < 0.01; S100B, *P* < 0.01; GDNF, *P* < 0.01, Additional file 2: Fig. S6G, H). However, overexpression of Glud1 was shown to upregulate the expression of glial markers (*P* < 0.05, Fig. 4D, E) by increasing monomethylation and decreasing trimethylation on H3K9 (*P* < 0.05, Fig. 4F, G) and H3K27 (*P* < 0.05, Fig. 4F, G). Besides, the ELISA results are consistent with the Western blot results (Fig. 4H, I), indicated that BMSCs treated with glutamate or overexpression of Glud1secrete more GDNF (*P* < 0.05) and S100B (*P* < 0.0001). And there was no significant difference in the mRNA expression level of Glud1 between OE-Glud1 BMSCs and OE-Glud1 BMSCs + Glu groups (*P* > 0.05, Fig. 4J). In addition, the genes associated with glial (GFAP: *P* < 0.05; GDNF: *P* < 0.05; S100B: *P* < 0.05), neurotrophic factor (BDNF: *P* < 0.05, Additional file 2: Fig. S7), and oxidative stress (COX5a: *P* < 0.05, SOD2: *P* < 0.05, Additional file 2: Fig. S8) were activated in glutamate-exposed groups and in OE-Glud1 BMSCs groups.

### Overexpression of Glud1 in BMSCs can significantly promote ENS regeneration in denervated mice

**Fig. 5:**
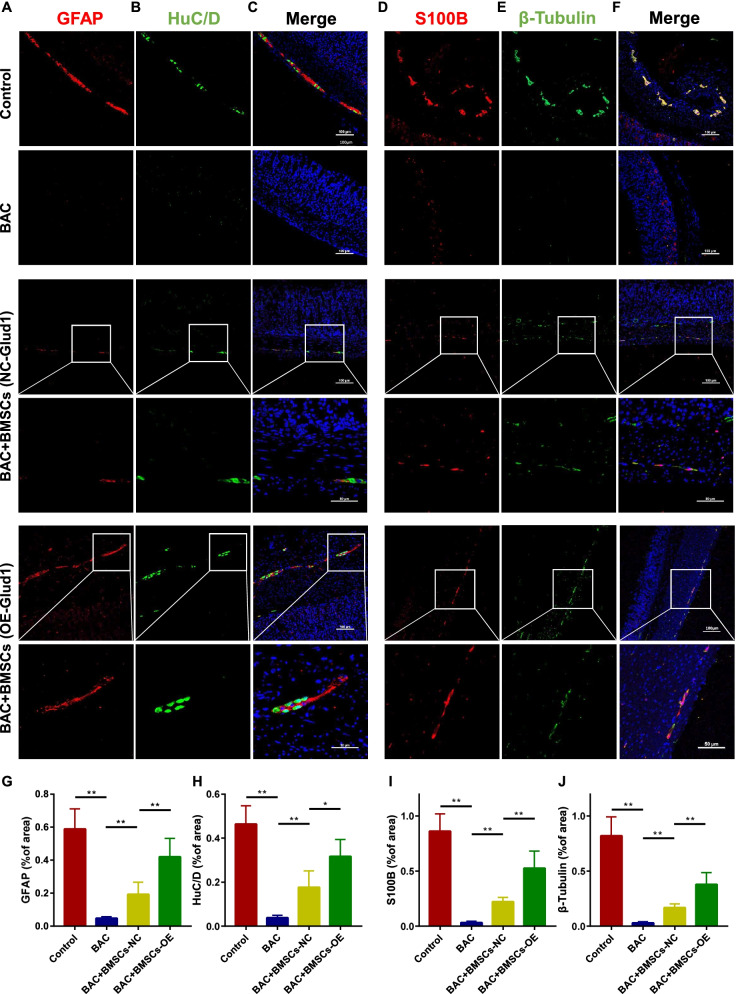
Effect of OE-glud1 BMSCs on the ENS regeneration in the BAC mice. **A** Representative immunofluorescence images in transverse sections of gastric tissue sections of GFAP (red) in each group. **B** Representative immunofluorescence images in transverse sections of gastric tissue sections of HuC/D (green) in each group. **C** Representative immunofluorescence images of GFAP (red) and HuC/D (green) in each group, the nuclei (blue). **D** Representative immunofluorescence images in transverse sections of gastric tissue sections of S100B (red) in each group. **E** Representative immunofluorescence images in transverse sections of gastric tissue sections of β-Tubulin (green) in each group. **F** Representative immunofluorescence images of S100B (red) and β-Tubulin (green) in each group, the nuclei (blue). **G**–**J** The statistics result of GFAP/HuC/D/S100B/β-Tubulin positive staining ratio to entire gastric mucosa sections in each group. Control: the control C57 mice; BAC: benzalkonium chloride-treated mice; BAC + BMSCs (NC-Glud1): BAC mice transplanted with BMSCs-NC; BAC + BMSCs (OE-Glud1): BAC mice transplanted with BMSCs-OE. Results were expressed as mean ± SD (*n* = 5 per group). *P < 0.05, **P < 0.01

**Fig. 6:**
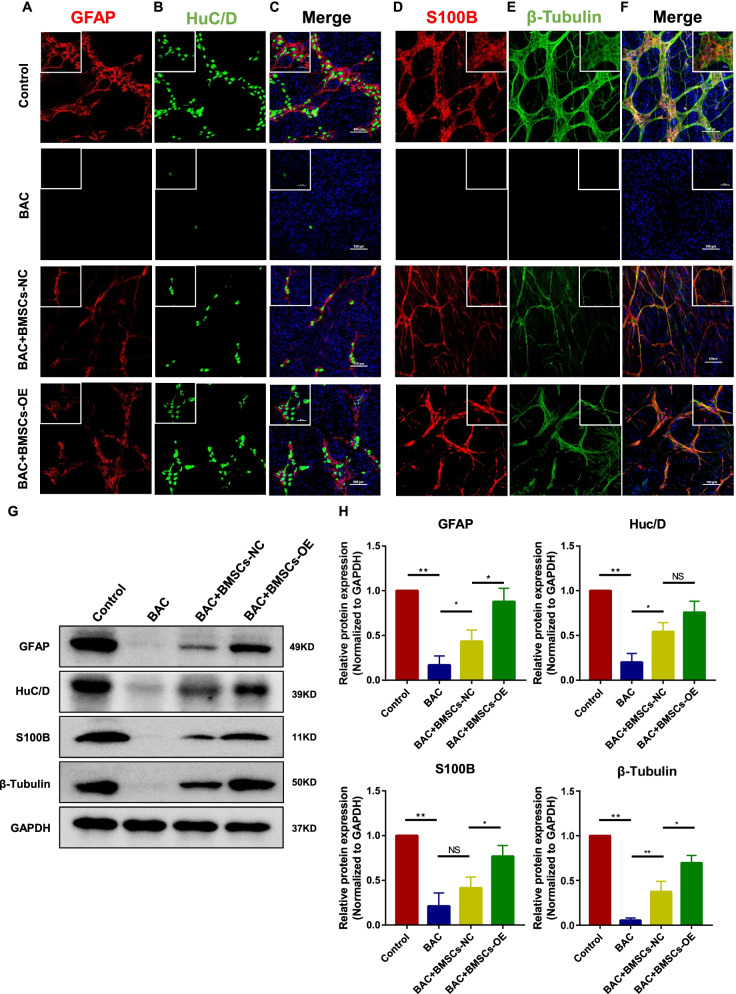
Effect of OE-glud1 BMSCs on the ENS regeneration in the BAC mice. **A** Representative immunofluorescence images in gastric myenteric plexus of GFAP (red) in each group. **B** Representative immunofluorescence images in gastric myenteric plexus of HuC/D (green) in each group. **C** Representative immunofluorescence images of GFAP (red) and HuC/D (green) in each group, the nuclei (blue). **D** Representative immunofluorescence images in gastric myenteric plexus of S100B (red) in each group. **E** Representative immunofluorescence images in gastric myenteric plexus of β-Tubulin (green) in each group. **F** Representative immunofluorescence images of S100B (red) and β-Tubulin (green) in each group, the nuclei (blue). **G** The protein expression of GFAP (Con:1, BAC: 0.17 ± 0.08, BAC + BMSCs-NC: 0.43 ± 0.10, BAC + BMSCs-OE: 0.88 ± 0.12)/HuC/D (Con: 1, BAC: 0.20 ± 0.08, BAC + BMSCs-NC: 0.54 ± 0.08, BAC + BMSCs-OE: 0.76 ± 0.10)/S100B (Con: 1, BAC: 0.21 ± 0.12, BAC + BMSCs-NC: 0.41 ± 0.10, BAC + BMSCs-OE: 0.77 ± 0.10)/β-Tubulin (Con: 1, BAC: 0.05 ± 0.02, BAC + BMSCs-NC: 0.37 ± 0.09, BAC + BMSCs-OE: 0.70 ± 0.07) in the gastric tissues was examined by immunoblotting. **H** The statistics results of GFAP/HuC/D/S100B/β-Tubulin protein expression in the gastric tissues. Control: the control C57 mice; BAC: benzalkonium chloride-treated mice; BAC + BMSCs (NC-Glud1): BAC mice transplanted with BMSCs-NC; BAC + BMSCs (OE-Glud1): BAC mice transplanted with BMSCs-OE. Results were expressed as mean ± SD (*n* = 4 per group). *P < 0.05, **P < 0.01, NS: no significance

The ENS-denervation model (BAC model) was used to determine whether the transplantation of OE-glud1 BMSCs could improve ENS regeneration. Results of the immunofluorescence assay of the transverse gastric sections and the myenteric plexus showed that the glial cells (GFAP/S100B, *P* < 0.01, Fig. 5G, I) and neuronal cells (HuC/D/β-Tubulin, *P* < 0.01, Fig. 5H, J) were significantly decreased in the BAC group compared with the control. Besides, regeneration of neurons and glial cells could be detected in the BMSCs transplantation group (Figs. 5, 6). In particular, there is a significantly increase in the number of regenerated neurons (HuC/D/β-Tubulin, *P* < 0.05, Fig. 5H, J) and glial cells (GFAP/S100B, *P* < 0.05, Fig. 5G, I) in the OE-glud1 BMSCs transplantation group compared with the NC-glud1 BMSCs transplantation group. In addition, immunofluorescence assay of the gastric myenteric plexus showed that the ENS network in the BAC + BMSCs (OE-glud1) group arranged more regular than the BAC + BMSCs (NC-glud1) group (Fig. 6A–F). Protein expression of GFAP/S100B/β-Tubulin was significantly upregulated in the BMSCs (OE-glud1) transplantation group than the BMSCs (NC-glud1) transplantation group (*P* < 0.05, Fig. 6G, H). Besides, BMSCs (OE-glud1) can significantly promote the repair of the ENS damaged microenvironment, including reducing the concentration of glutamate (*P* < 0.05, Additional file 2: Fig. S1B) and downregulating the levels of inflammatory factors (TNF-α: *P* < 0.05, IL-6: *P* < 0.0001, Additional file 2: Fig. S1C–E). These results suggest that BMSCs overexpressing Glud1 had a strong promotion effect on ENS regeneration in denervated mice.

### BMSCs (OE-Glud1) can significantly promote ENS regeneration by upregulating glial cell protein expression and histone demethylation level

**Fig. 7:**
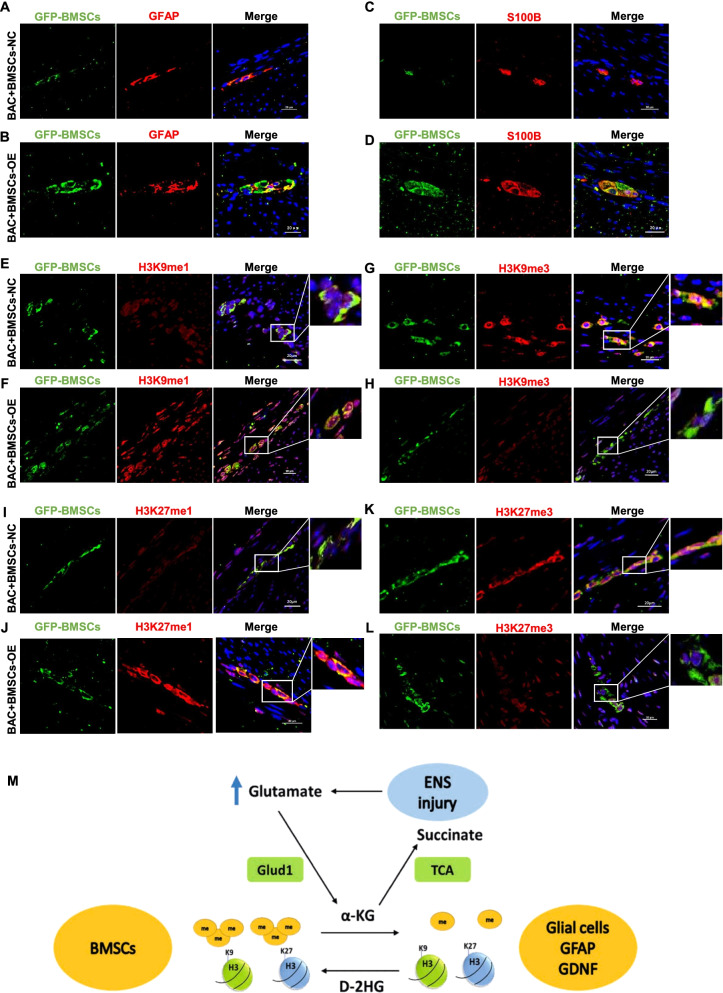
BMSCs (OE-Glud1) expressing higher glial cell characteristics markers and histone demethylation level than BMSCs (NC-Glud1) to promote ENS regeneration. **A**–**D** GFP-labeled BMSCs-NC/BMSCs-OE (green) and GFAP/S100B (red) were jointly immunostained in the transverse sections of gastric, the nuclei were labeled with DAPI (blue). **E**–**L** GFP-labeled BMSCs-NC/BMSCs-OE (green) and H3K9me1/H3K9me3/H3K27me1/H3K27me3 (red) were jointly immunostained in the transverse sections of gastric, the nuclei were labeled with DAPI (blue). **M** The cartoon of the mechanism that BMSCs promoting ENS regeneration. Glud1 hydrolysis metabolite glutamate affects a-KG, which further affects H3K9me3 and H3K27me3 levels and the expression of GFAP, S100B, and GDNF, which alters the BMSCs’ glial cell properties. These results are representative of at least three times independent experiments. BAC + BMSCs (NC-Glud1): BAC mice transplanted with BMSCs-NC; BAC + BMSCs (OE-Glud1): BAC mice transplanted with BMSCs-OE. ENS: enteric nervous system; Glud1: glutamate dehydrogenase 1; TCA: tricarboxylic acid; α-KG: alpha-ketoglutarate, D-2HG: D-2-hydroxyglutarate

To further verify the mechanism that BMSCs promoting ENS regeneration in the ENS-injured microenvironment, immunofluorescence assay of the transverse gastric sections was performed to trace GFP-labeled BMSCs (GFP-BMSCs) in the myenteric plexus. The results of immunofluorescence double staining co-localization showed that the expression of glial cell characteristic markers (GFAP/S100B) for BMSCs (OE-Glud1) was higher than BMSCs (NC-Glud1) (Fig. 7A–D). In addition, immunostaining of GFP-BMSCs in combination with histone-methylated protein showed increased expression of H3K9me1 and H3K27me1 and decreased expression of H3K9me3 and H3K27me3 in BMSCs (OE-Glud1) group compared with the BMSCs (NC-Glud1) group (Fig. 7E–L). These results indicated that BMSCs overexpressing Glud1 had a strong promotion effect on ENS regeneration in denervated mice by increasing monomethylation and decreasing trimethylation on H3K9 and H3K27, and upregulating glial cell protein expression.

## Discussion

Previous studies showed that transplantation of BMSCs was effective in the treatment of gastrointestinal motility disorders [6, 7]. However, how cellular energy metabolism regulates the fate of BMSCs in the ENS-injured high-glutamate microenvironment is unclear. To the best of our knowledge, this study was the first to demonstrate that the glial cell characteristics protein of BMSCs were significantly upregulated in high-glutamate microenvironment. And BMSCs overexpressing Glud1 can effectively promote the regeneration of enteric neurons and the remodeling of ENS by increasing histone demethylation and upregulating the expression of glial cell protein.

This study showed significantly increased glutamate concentration in the ENS injury microenvironment, consistent with previous research [28]. Excessive glutamate may lead to neuronal injuries and neurodegeneration [29]. Researches have reported that mesenchymal stem cells (MSCs) can mediate protection in neurons by regulating energy metabolism (high glutamate) [30, 31]. However, only a few studies have investigated the effects of glutamate on the biological characteristics of the BMSCs. This study showed that the expression of glial cell characteristic proteins (GFAP/GDNF/S100B) in BMSCs was significantly upregulated in homoglutamate microenvironment. It is reported that gene activation and protein expression of GFAP play an important role in astroglia cell repair following central nervous system dysfunction and degeneration [32]. Glial cell-derived neurotrophic factor (GDNF) and S100B are mainly secreted by glial cells and are important for the survival, maintenance, and regeneration of specific neuronal populations [33]. In this study, the increased expression of characteristic glial cell proteins of BMSCs in the homoglutamate microenvironment was shown to be the basis for promoting ENS regeneration.

Glutamate has been shown to be converted to α-KG in the TCA cycle. α-KG is a substrate of dioxygenases that participates in cell methylation and various cell activities [34]. For example, Kang et al*.* reported that α-KG regulates the differentiation and function of brown fat cells by regulating histone methylation [35]. Besides, Hwang et al*.* reported that α-KG maintains the pluripotency and self-renewal ability of embryonic stem cells by regulating histone methylation levels [36]. In the present study, increased α-KG levels in the BMSCs were shown to reduce trimethylation of H3K9 and H3K27. Histone methylation plays a regulatory role in animal development [37]. Studies have shown that H3K9 and H3K27 histone methylation is closely related to neurodevelopmental disorders [38]. Lin et al*.* reported that the histone H3 lysine 9 demethylase KDM3A facilitates accessibility of the *Xenopus* Neurog2 chromatin during neuronal transcription [16]. This study showed that inhibition of histone demethylation in BMSCs could downregulate the protein expression of characteristic glial cell marker.

Glutamate dehydrogenase 1 (Glud1) is a key enzyme in glutaminolysis that converts glutamate to α-KG. It is reported that Glud1 degradation can decrease the activity of α-KG-dependent lysine demethylases (KDMs). Reduced KDM activity further leads to increased histone H3 lysine 9 and 27 methylation to support cell survival [39]. According to this study, the overexpression of Glud1 in BMSCs increased monomethylation and decreased trimethylation on H3K9 and H3K27 to upregulate the expression of characteristic glial cell proteins.

Glia cells constitute at least half of the mammalian nervous system cells. They are crucial regulators of the nervous system and control the development, plasticity, and diseases of the nervous system. An important role is to respond to nerve damage, a complex change known as reactive gliosis. Another function is to serve as stem cells, to promote nerve regeneration in normal and disease [40]. Glial cells secrete various neurotrophic factors, such as GDNF. Soret et al*.* reported that GDNF induced enteric neurogenesis and improved the structure and function of the colon in Hirschsprung disease mouse models [41]. In addition, previous studies have also shown that high-frequency electroacupuncture at ST-36 acupoints promotes the regeneration of enteric neurons in diabetic rats by promoting secretion of GDNF [42]. In this study, overexpression of Glud1 in BMSCs upregulated the expression of the glial cell markers (GFAP) and neurotrophic factors (GDNF).

BMSCs show the ability for self-renewal and differentiation into various cell types. In addition, BMSCs secrete growth factors and anti-apoptotic factors, which have the potential for tissue repair and regeneration [43]. For example, transplantation of BMSCs was shown to promote remyelination and regeneration of damaged central axons in hemisections and cross sections of spinal cord injury models [44]. Besides, the brain-derived neurotrophic factor (BDNF) and GDNF produced by the transplanted BMSCs could synergistically promote peripheral nerve repair [45]. For gastrointestinal motility disorders, Mazzanti et al*.* reported that the transplantation of BMSCs improved the contractility of the LES sphincter, thus preventing gastro-esophageal reflux [46]. Further, previous studies also demonstrated that the transplantation of BMSCs promoted the regeneration of gastric nerves in denervated mice and the ENS remodeling in diabetic mice [6, 7]. In this study, regenerated neurons and glial cells can be detected in the BAC group after transplantation of BMSCs, consistent with previous studies. In addition, the ENS network was significantly improved in the BMSCs (OE-glud1) transplantation group compared with the BMSCs (NC-glud1). These findings suggest that BMSCs (OE-glud1) promoted the conversion of glutamate to α-KG in the ENS injury microenvironment, upregulated histone demethylation, and the differentiation of BMSCs. Further, the “Glial-like” BMSCs (OE-glud1) secreted more GDNF and S100B to support neuronal growth and promote ENS remodeling.

## Conclusions

In conclusion, this study demonstrated that BMSCs overexpressing Glud1 significantly promote the ENS regeneration by increasing histone demethylation on H3K9 and H3K27 and upregulating the expression of glial cell protein in ENS injury high-glutamate microenvironment. Genomic modification of BMSCs promotes ENS remodeling and provides a basis for developing highly effective therapies for managing gastrointestinal neuropathy.

## Supplementary Information

**Additional file 1: Table S1.** Primers used for qRT-PCR and ChIP-qPCR.

**Additional file 2: Fig. S1.** The relative glutamate concentration, the expression levels of inflammatory cytokines (IL-1β/TNF-α/IL-6) and anti-inflammatory cytokines (IL-13/IL-10/IL-4) of gastric tissue in different groups of mice. **A** and **B** Mouse stomach tissue presents a high-glutamate microenvironment after nerve injury, and BMSCs transplantation reduced the glutamate concentration. **C**–**H** The transcripts of inflammatory and anti-inflammatory cytokines were determined by RT-PCR assay. The inflammatory cytokines (TNF-α/IL-6) and anti-inflammatory (IL-13/IL-10/IL-4) were increased in ENS injury mice compared with control, and BMSCs transplantation reduced the level of inflammatory cytokines (TNF-α/IL-6), increased the level of anti-inflammatory (IL-13). BAC: benzalkonium chloride-treated mice; BAC + BMSCs (NC-Glud1): BAC mice transplanted with BMSCs-NC; BAC + BMSCs (OE-Glud1): BAC mice transplanted with BMSCs-OE; ENS: enteric nervous system. Results were expressed as mean ± SD. *P < 0.05, **P < 0.01, ****P < 0.0001, NS: no significance. **Fig. S2.** Glutamate increases the expression of glial cell characteristic protein for BMSCs. Different concentrations of glutamate (0, 0.2 mM, 0.5 mM, 1 mM, 2 mM, 4 mM) were incubated with BMSCs for 24 or 48 h. **A**–**D** Representative immunoblot bands and histogram of relative expression for the GFAP and GDNF proteins. GAPDH was used as a loading control. **E** and **F** Fluorescent label-CFSE proliferation detection of BMSCs and statistical analysis. Glu: Glutamate; CFSE: carboxyfluorescein diacetate succinimidyl ester. These results are representative of at least three times independent experiments. *P < 0.05, **P < 0.01, NS: no significance. **Fig. S3.** The GABA receptors of BMSCs is activated under glutamate intervention. **A** and **B** The transcripts of GABARA and GABARB genes of BMSCs treated with glutamate were determined by a RT-PCR assay, and the GABARA genes of BMSCs is activated with glutamate intervention. ***P < 0.001, NS: no significance. **Fig. S4.** The mesenchymal genes of BMSCs is activated under glutamate intervention. **A** and **B** The transcripts of mesenchymal genes (Snail and Twist) in BMSCs treated with glutamate were determined by a RT-PCR assay. **C**–**E** The transcripts of genes involved in cell migration (MMP2, MMP9 and CXCR4) of BMSCs treated with glutamate were determined by a RT-PCR assay, and CXCR 4 of BMSCs is activated under glutamate intervention (P < 0.05). *P < 0.05, **P < 0.01, ****P < 0.0001, NS: no significance. **Fig. S5.** The intracellular α-KG, succinate concentration and α-kg/succinate ratio of BMSCs. BMSCs were incubated with D-2HG (2 mM) or Glu (2 mM) for 24 h. The intracellular α-KG content and the ratio of α-KG/succinate of BMSCs were significantly increased under glutamate intervention, and D-2HG reversed this change. Glu: Glutamate; D-2HG: D-2-hydroxyglutaric acid. These results are representative of at least three times independent experiments. *P < 0.05, **P < 0.01, ***P < 0.001, NS: no significance. **Fig. S6.** Knockdown of Glud1 derived from the BMSCs could resulted in decreased expression of the characteristic glial cell proteins. **A** Transfection of recombinant lentiviral vectors into BMSCs and resultant changes in Glud1 expression. Fluorescence microscope patterns of BMSCs-NC, BMSCs-KD and BMSCs-OE. **B** and **C** Representative immunoblot bands and histogram of relative expression Glud1 protein in BMSCs-NC, BMSCs-KD and BMSCs-OE. **D–F** The intracellular α-KG, succinate concentration and α-kg/succinate ratio of BMSCs. The intracellular α-KG content and the ratio of α-KG/succinate of BMSCs were significantly increased under glutamate intervention, and knockdown of Glud1 derived from the BMSCs reversed this change. **G** and **H** Representative immunoblot bands and histogram of relative expression of for the GFAP, S100B, and GDNF in each group. GAPDH was used as a loading control. Glu: Glutamate; Glud1: Glutamate Dehydrogenase 1; OE: overexpression; KD: knockdown; NC: negative control. These results are representative of at least three times independent experiments. *P < 0.05, **P < 0.01, ***P < 0.001, ****P < 0.0001, NS: no significance. **Fig. S7.** The genes associated with glial cell properties of BMSCs is activated under glutamate intervention and in OE-Glud1 BMSCs. **A** and **B** The transcripts of neuronal genes (PGP9.5 and β-tubulin) of BMSCs in different groups were determined by a RT-PCR assay. **C**–**E** The transcripts of glial related genes (GFAP, GDNF, S100B) of BMSCs in different groups were determined by a RT-PCR assay. **F** and **G** The transcripts of neurotrophic factor genes (BDNF, NGF) of BMSCs in different groups were determined by a RT-PCR assay. **H** The transcripts of cell proliferation-related genes (PCNA) of BMSCs in different groups were determined by a RT-PCR assay. **I** The transcripts of cell apoptosis related genes (Caspase-1) of BMSCs in different groups were determined by a RT-PCR assay. *P < 0.05, **P < 0.01, NS: no significance. **Fig. S8.** The genes associated with oxidative stress of BMSCs is activated under glutamate intervention and in OE-Glud1 BMSCs. **A–C** The transcripts of oxidative stress related genes (COX5a, SOD2, GPX4) in BMSCs treated with glutamate were determined by a RT-PCR assay. COX5a/SOD2 of BMSCs is activated under glutamate intervention and in OE-Glud1 BMSCs. *P < 0.05, **P < 0.01, NS: no significance.

## Data Availability

The raw data generated and analyzed in the current study are not publicly available but are available from the corresponding author on reasonable request.

## Abbreviations

BMSCs: Bone marrow-derived mesenchymal stem cells
ENS: Enteric nervous system
BAC: Benzalkonium chloride
PBS: Phosphate-buffered saline
TCA: Tricarboxylic acid
Glu: Glutamate
DM-α-KG: Dimethyl-alpha-KG
α-KG: α-Ketoglutarate
Suc: Succinate
D-2HG: D-2-hydroxyglutarate
Glud1: Glutamate dehydrogenase 1
KD: Knockdown
OE: Overexpression
NC: Negative control
GFP: Green fluorescent protein
GFAP: Glial fibrillary acidic protein
GDNF: Glial cell line-derived neurotrophic factor
GAPDH: Glyceraldehyde-3-phosphate dehydrogenase
RT-PCR: Reverse transcription-polymerase chain reaction
ChIP: Chromatin immunoprecipitation
