# Supplementary material for: Glutamate regulates gliosis of BMSCs to promote ENS regeneration through α-KG and H3K9/H3K27 demethylation
Source: Stem Cell Res Ther. 2022 Jun 17;13:255. doi: 10.1186/s13287-022-02936-7 (PMC9205030; doi:10.1186/s13287-022-02936-7)
Supplement: Supplementary file 1 — Additional file 1: Table S1. Primers used for qRT-PCR and ChIP-qPCR. [file 13287_2022_2936_MOESM1_ESM.docx]

Supplementary Table 1. Primers used for qRT-PCR and ChIP-qPCR

| **Number** | Primer for RT-PCR**（**mouse**）** | Forward primer | Reverse primer |
| --- | --- | --- | --- |
| 1 | Glud1 | CTGTGGTCGATGTACCGTTTG | AGCTCCATAGTGAACCTCCGT |
| 2 | IL-1β | CATCTTCTCAAAATTCGAGTGACAA | TGGGAGTAGACAAGGTACAACCC |
| 3 | IL-6 | CTGCAAGAGACTTCCATCCAG | AGTGGTATAGACAGGTCTGTTGG |
| 4 | TNF-α | CATCTTCTCAAAATTCGAGTGACAA | TGGGAGTAGACAAGGTACAACCC |
| 5 | IL-10 | GCTGGACAACATACTGCTAACC | GCTGGACAACATACTGCTAACC |
| 6 | IL-13 | GTATGGAGTGTGGACCTGGC | TTTTGGTATCGGGGAGGCTG |
| 7 | IL- 4 | TCACAGCAACGAAGAACACCA | CAGGCATCGAAAAGCCCGAA |
| 8 | GABARA | GCCCCGAGGTAAGTGAGACT | GCTCGCTTCCCTGCTAGAAC |
| 9 | GABARB | TGACCCTGGAAAATGGGAAGG | TGTGGCGTTCGATTCACCT |
| 10 | Snail | ATGGAGTGCCTTTGTACCCG | CAGTAACCACCCTGCTGAGG |
| 11 | Twist | ACAAGAATCAGGGCGTGGG | GGGGGACACAAACGAGTGTT |
| 12 | MMP2 | CCCCATGAAGCCTTGTTTACC | CAGTGGACATAGCGGTCTCG |
| 13 | MMP9 | CGACTTTTGTGGTCTTCCCCA | TGCTTCTCTCCCATCATCTGG |
| 14 | CXCR4 | GTAACCACCACGGCTGTAGA | TGCCGACTATGCCAGTCAAG |
| 15 | PGP 9.5 | AGATGCTGAACAAAGTGTTGGC | AGGGCTAACTTCCTGTCCCT |
| 16 | β-tubulin | GCGGCAACTATGTAGGGGAC | AGCACCACTCTGACCAAAGA |
| 17 | GFAP | GCGAAGAAAACCGCATCACC | AAGGGAGAGCTGGCAGG |
| 18 | GDNF | CGTCATCAAACTGGTCAGGA | CGCTGAACCACTCCCTC |
| 19 | S100B | GATGTCCGAGCTGGAGAAGG | CCTGCTCCTTGATTTCCTCCA |
| 20 | NGF | TCTGTGTACGGTTCTGCCTG | CAGCTTTCTATACTGGCCGC |
| 21 | BDNF | AGCAGAGTCCATTCAGCACC | TCTGACTCTCTCTCCAGCCC |
| 22 | PCNA | GGGTTGGTAGTTGTCGCTGT | CCAAGGAGACGTGAGACGAG |
| 23 | Caspase1 | ACTGACTGGGACCCTCAAGT | GCAAGACGTGTACGAGTGGT |
| 24 | SOD2 | AGGAGAGTTGCTGGAGGCTA | TCTGTAAGCGACCTTGCTCC |
| 25 | GPX4 | ACCTGGACGCCAAAGTCCTA | GTGACGATGCACACGAAACC |
| 26 | GAPDH | AGGAGCGAGACCCCACTAACA | AGGGGGGCTAAGCAGTTGGT |

| **Number** | Primer for ChIP-qPCR | Forward primer | Reverse primer |
| --- | --- | --- | --- |
| 1 | GFAP | CCAGGTCCCCTGGTCTTTAC | TCTGAGACTAGCAGAGCACAG |
| 2 | GDNF | TGTATGTGCCTACCCCACG | GGTCTCCAAATATCTGCTGGCT |
| 3 | S100B | AAAGGTGCAGAGAAGCTGACT | CCAATGGGGGCCAAGGATAG |
