# Supplementary material for: Glutamate regulates gliosis of BMSCs to promote ENS regeneration through α-KG and H3K9/H3K27 demethylation
Source: Stem Cell Res Ther. 2022 Jun 17;13:255. doi: 10.1186/s13287-022-02936-7 (PMC9205030; doi:10.1186/s13287-022-02936-7)

Supplementary Figure 1

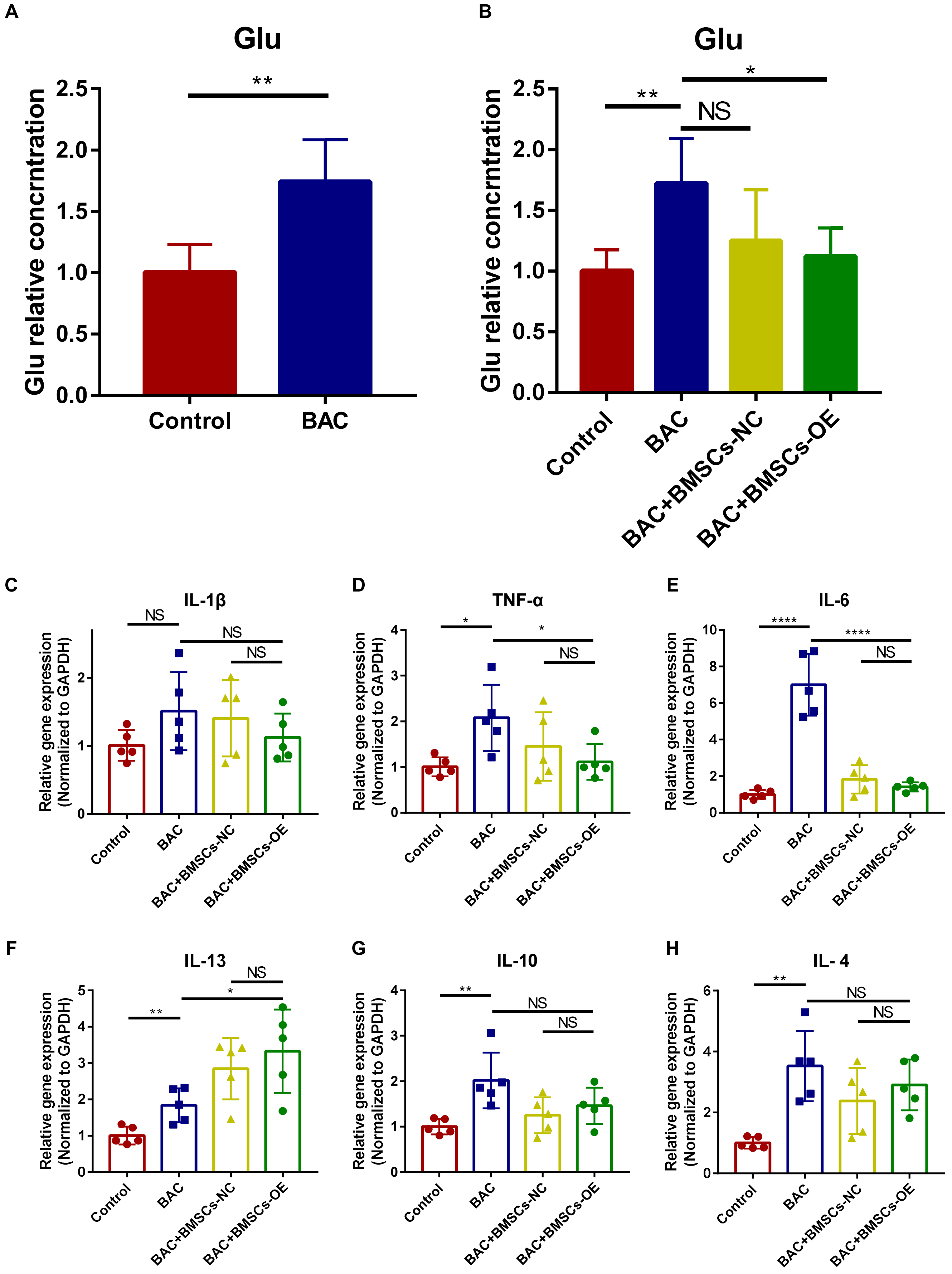

Supplementary Figure 2

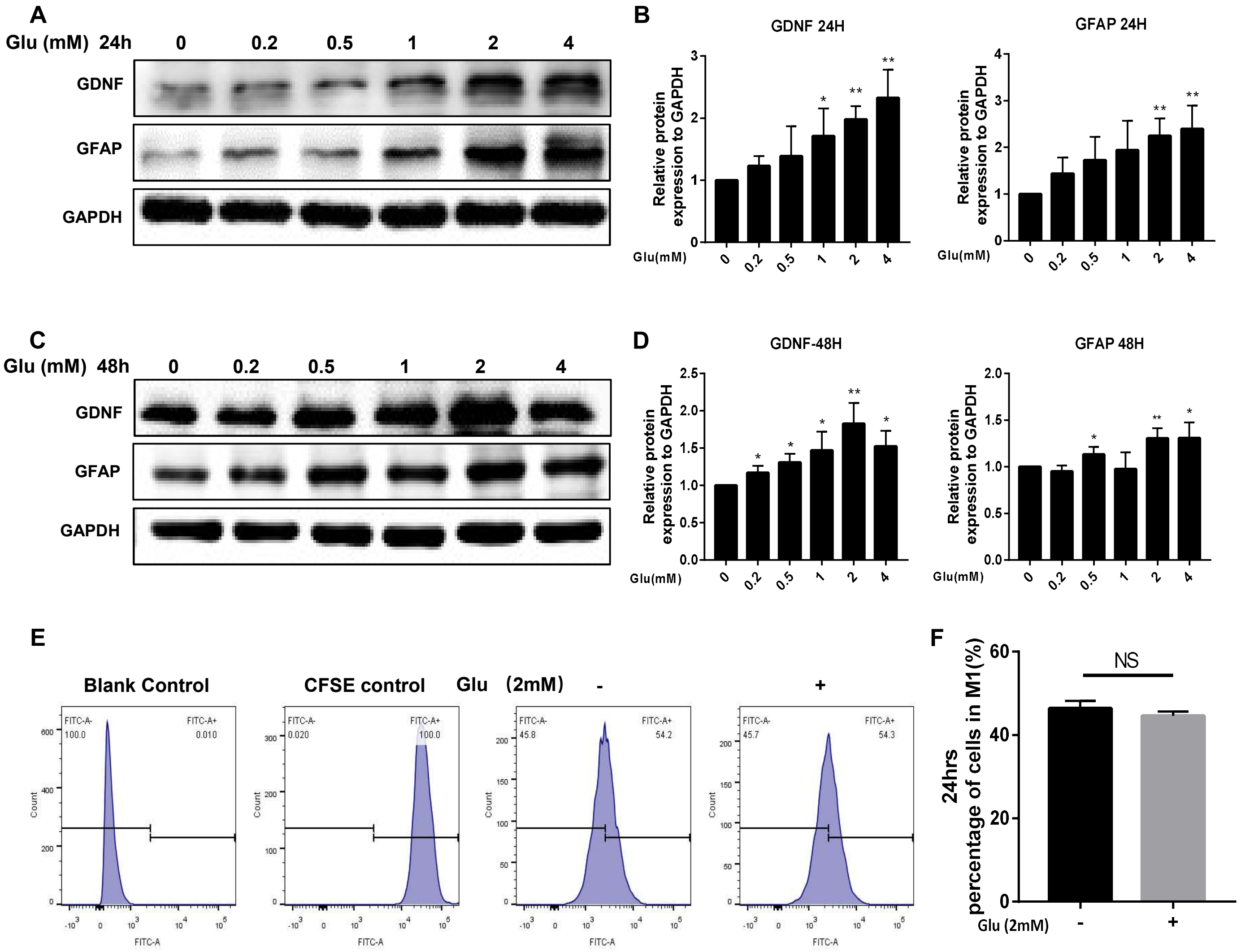

Supplementary Figure 3

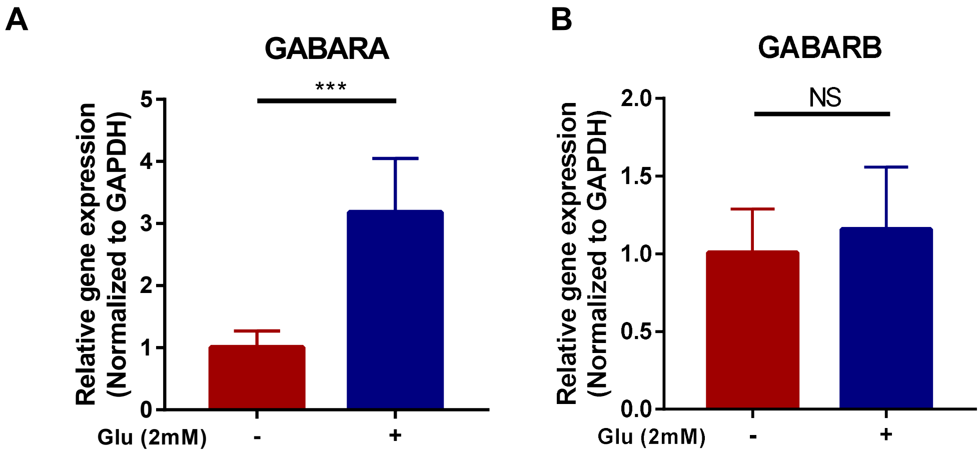

Supplementary Figure 4

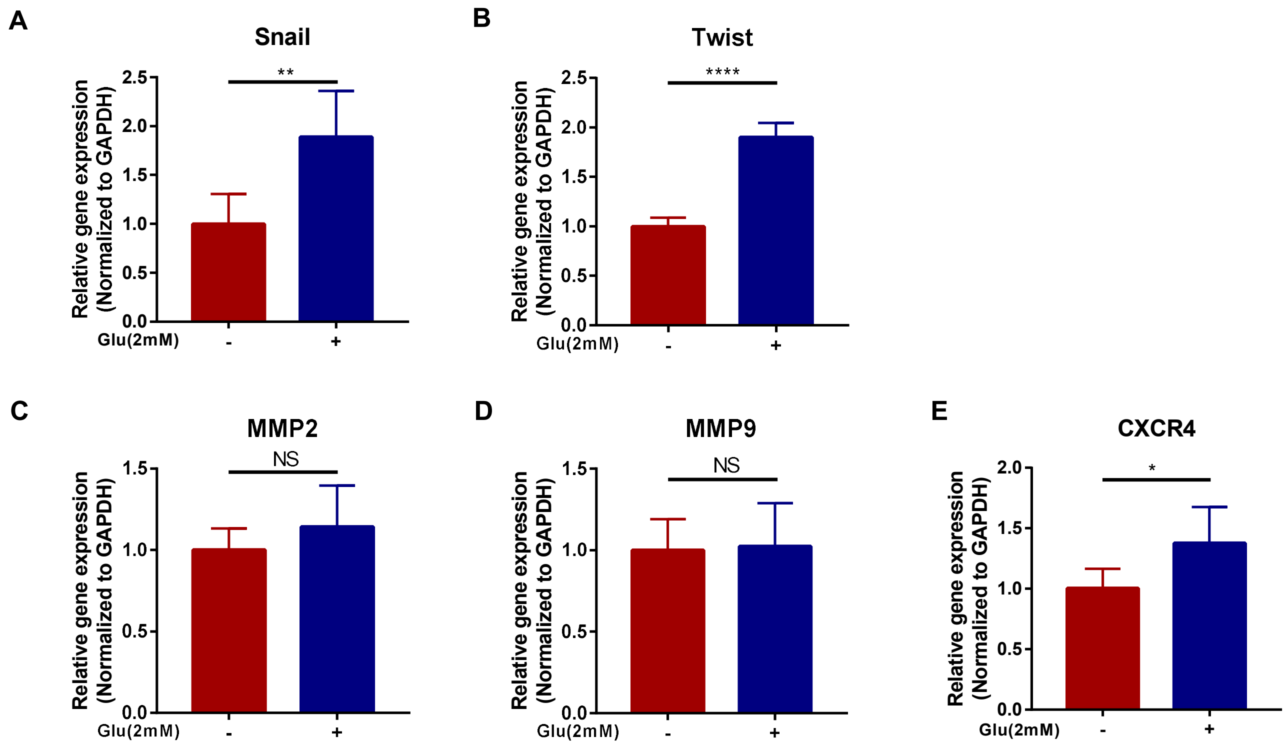

Supplementary Figure 5

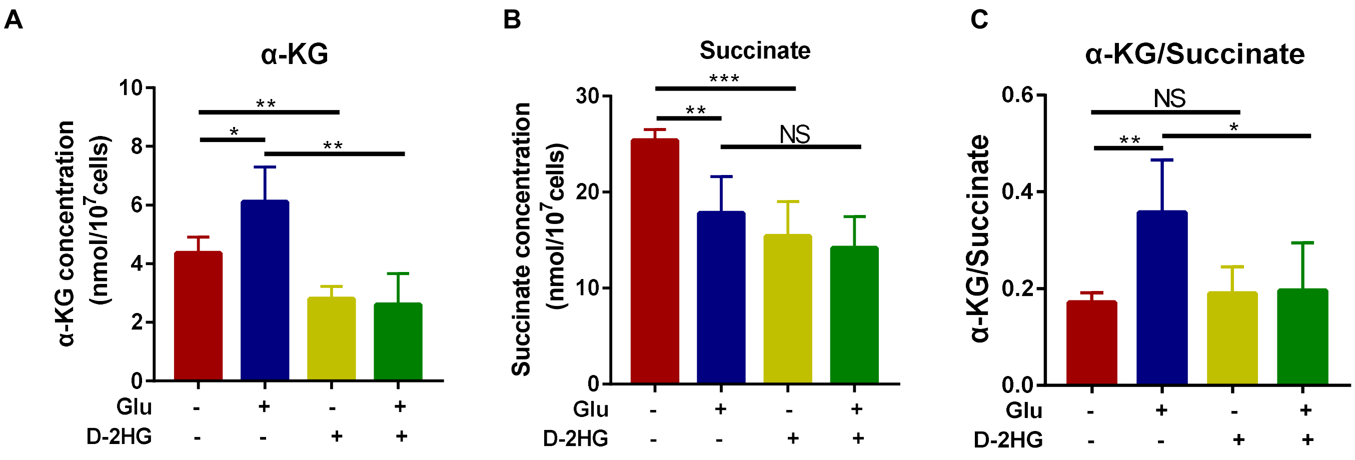

Supplementary Figure 6

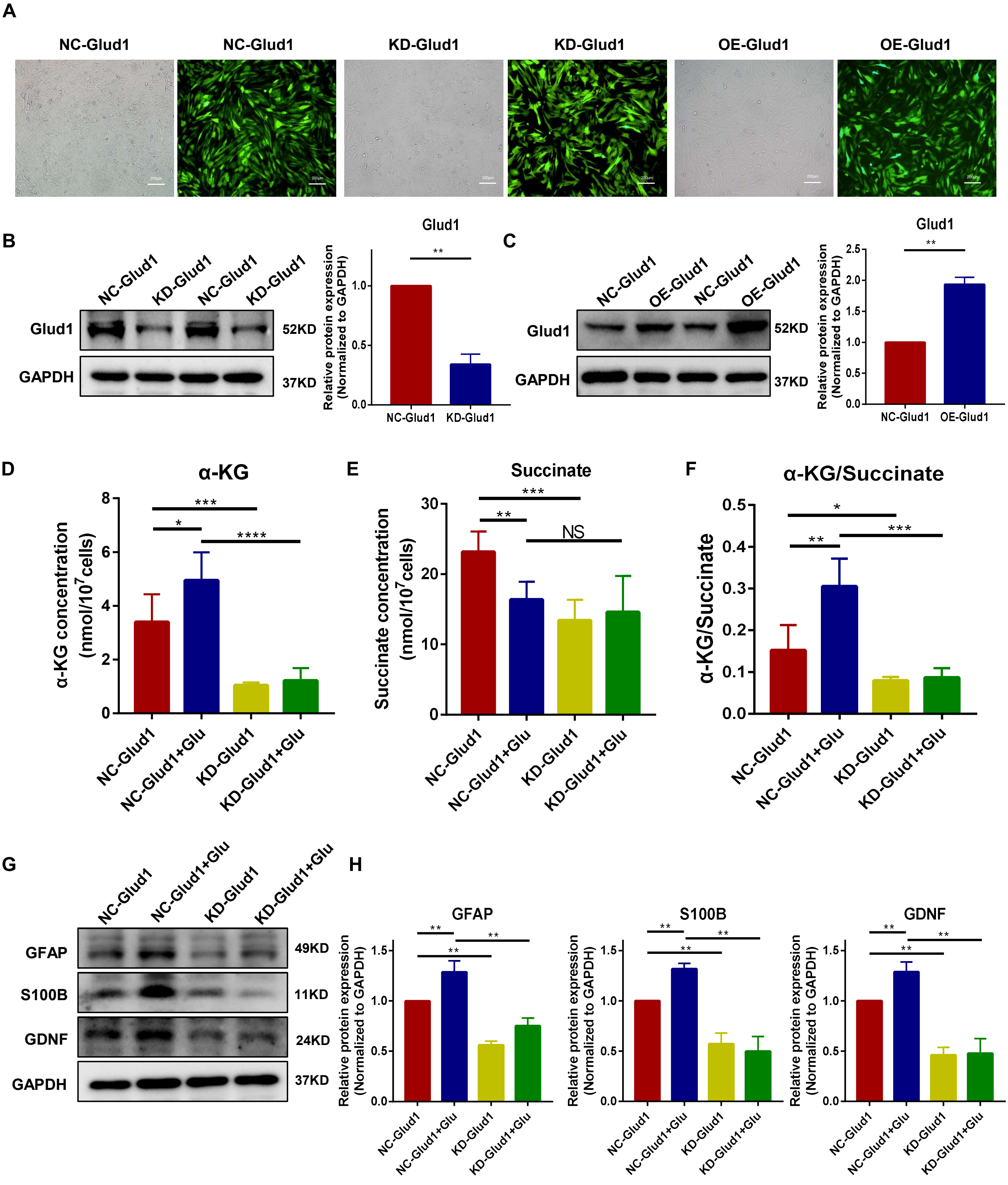

Supplementary Figure 7

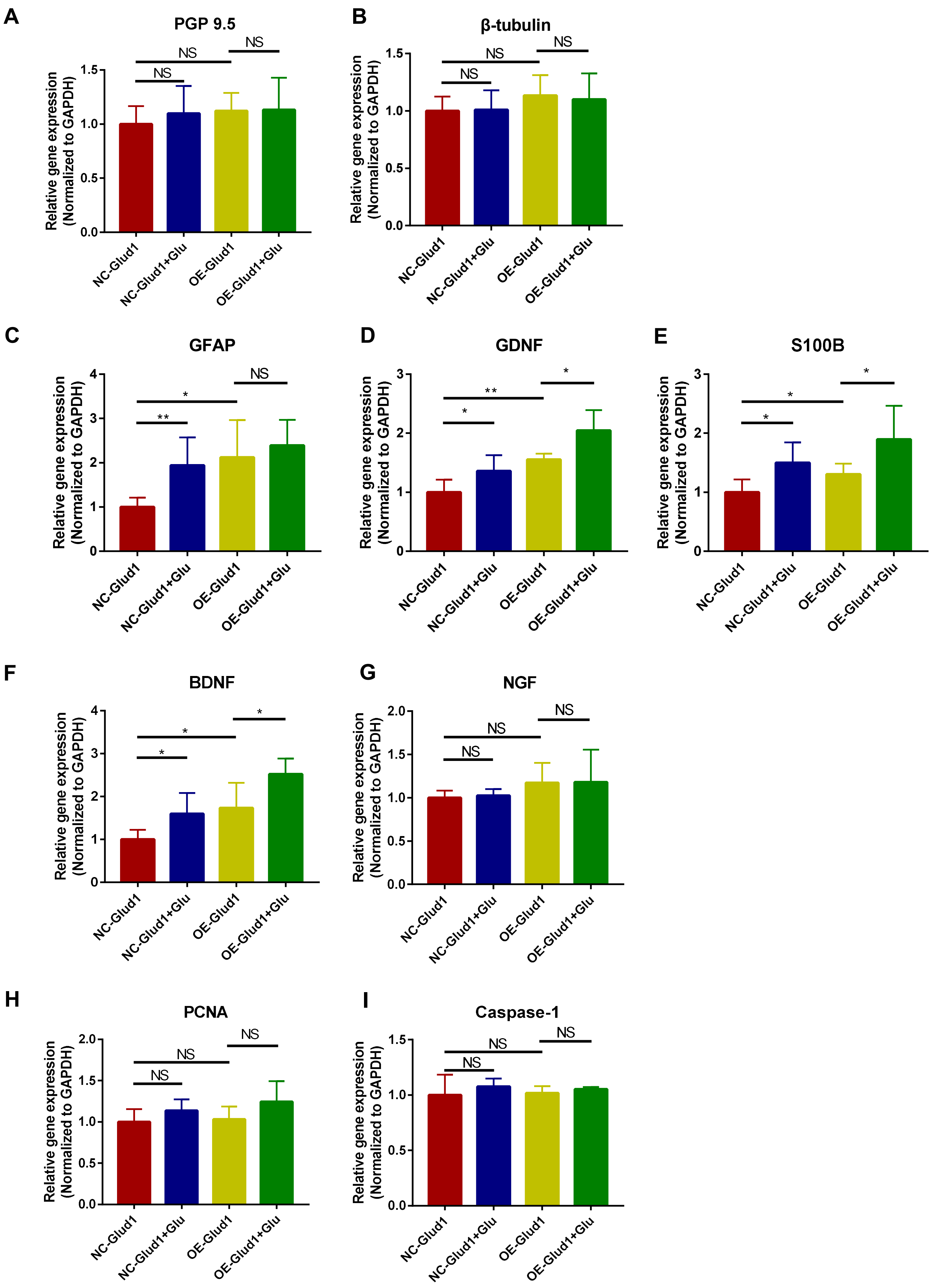

Supplementary Figure 8

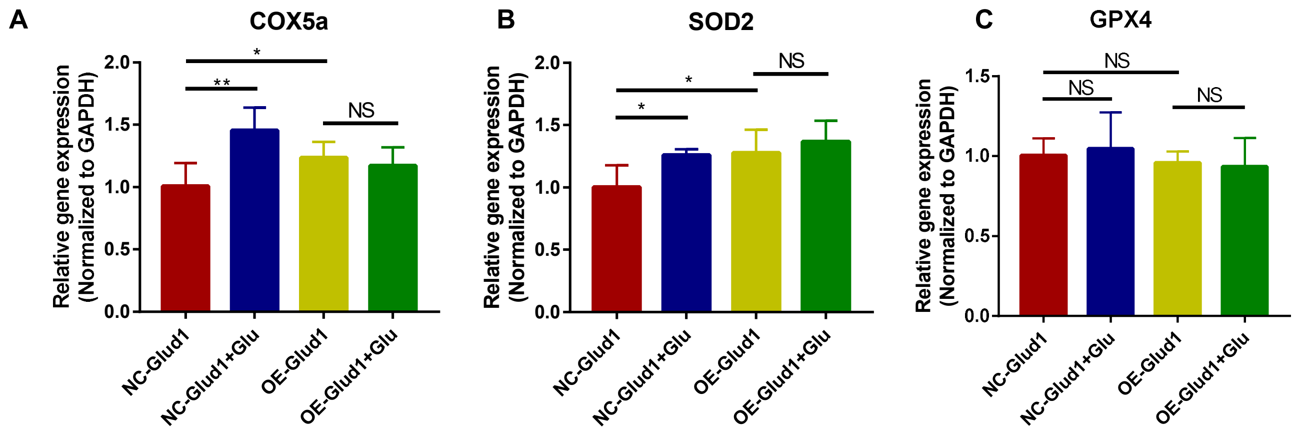

Supplement: Supplementary file 2 — Additional file 2: Fig. S1. The relative glutamate concentration, the expression levels of inflammatory cytokines (IL-1β/TNF-α/IL-6) and anti-inflammatory cytokines (IL-13/IL-10/IL-4) of gastric tissue in different groups of mice. A and B Mouse stomach tissue presents a high-glutamate microenvironment after nerve injury, and BMSCs transplantation reduced the glutamate concentration. C–H The transcripts of inflammatory and anti-inflammatory cytokines were determined by RT-PCR assay. The inflammatory cytokines (TNF-α/IL-6) and anti-inflammatory (IL-13/IL-10/IL-4) were increased in ENS injury mice compared with control, and BMSCs transplantation reduced the level of inflammatory cytokines (TNF-α/IL-6), increased the level of anti-inflammatory (IL-13). BAC: benzalkonium chloride-treated mice; BAC + BMSCs (NC-Glud1): BAC mice transplanted with BMSCs-NC; BAC + BMSCs (OE-Glud1): BAC mice transplanted with BMSCs-OE; ENS: enteric nervous system. Results were expressed as mean ± SD. *P < 0.05, **P < 0.01, ****P < 0.0001, NS: no significance. Fig. S2. Glutamate increases the expression of glial cell characteristic protein for BMSCs. Different concentrations of glutamate (0, 0.2 mM, 0.5 mM, 1 mM, 2 mM, 4 mM) were incubated with BMSCs for 24 or 48 h. A–D Representative immunoblot bands and histogram of relative expression for the GFAP and GDNF proteins. GAPDH was used as a loading control. E and F Fluorescent label-CFSE proliferation detection of BMSCs and statistical analysis. Glu: Glutamate; CFSE: carboxyfluorescein diacetate succinimidyl ester. These results are representative of at least three times independent experiments. *P < 0.05, **P < 0.01, NS: no significance. Fig. S3. The GABA receptors of BMSCs is activated under glutamate intervention. A and B The transcripts of GABARA and GABARB genes of BMSCs treated with glutamate were determined by a RT-PCR assay, and the GABARA genes of BMSCs is activated with glutamate intervention. ***P < 0.001, NS: no significance. Fig. S4. [file 13287_2022_2936_MOESM2_ESM.pdf]
